# PHYSICAL RESTRAINTS AT HOME AND THEIR FACTORS: A ONE-YEAR LONGITUDINAL SURVEY AMONG HOME CARE SERVICE USERS IN JAPAN

**DOI:** 10.1093/geroni/igac059.1361

**Published:** 2022-12-20

**Authors:** Noriko Yamamoto-Mitani, Hanako Numata, Chie Fukui

**Affiliations:** The University of Tokyo, Tokyo, Tokyo, Japan; The University of Tokyo, Tokyo, Tokyo, Japan; The University of Tokyo, Tokyo, Tokyo, Japan

## Abstract

We conducted a one-year survey of homecare service users aged 75 years and older. Data from 769 clients were examined. Altogether 47 (6.1%) received physical restraint at least once among the five survey points conducted over a one-year period. Among them, 27 (57.4%) received restraint only at one point, and only 2 received restraint in all five points. In the bivariate analyses, the factors associated with the use of physical restraint were: diagnosis of neurological disease, unstable general condition or terminal stage, high level of care required, medical treatment, and family burden. When those who were released from the restraint at 12 months (n=30) were compared with those who were not (n=17), not having stroke, not receiving suctioning, and not having home-visit medical care were significant factors. Physical restraints at home were not common but we must be careful when we serve clients with significant factors of prolonged restraints.

